# The localization of HER4 intracellular domain and expression of its alternately-spliced isoforms have prognostic significance in ER+ HER2- breast cancer

**DOI:** 10.18632/oncotarget.2002

**Published:** 2014-05-28

**Authors:** Saori Fujiwara, Mutsuko Yamamoto-Ibusuk, Yutaka Yamamoto, Satoko Yamamoto, Mai Tomiguchi, Takashi Takeshita, Mitsuhiro Hayashi, Aiko Sueta, Hirotaka Iwase

**Affiliations:** ^1^ Department of Breast and Endocrine Surgery, Graduate School of Medical Sciences, Kumamoto University, Honjo, Chuo-ku, Kumamoto 860-8556, Japan; ^2^ Department of Molecular Targeting Therapy for Breast Cancer, Graduate School of Medical Sciences, Kumamoto University, Honjo, Chuo-ku, Kumamoto 860-8556, Japan

**Keywords:** HER4, 4ICD, Splicing variants, Breast cancer, Endocrine therapy, Prognosis

## Abstract

Human epidermal growth factor receptors (HERs) are known to play a pivotal role in breast cancer, both as prognostic markers and as therapeutic targets. The importance of Her4 expression is, however, still controversially discussed; there are few reports on the clinical significance of HER4, its splice variants, and cleaved HER4 intracellular domains (4ICD) which function differently depending on their localization in breast cancer. In 238 primary invasive breast cancer patients, we analyzed the expression levels of HER4 extracellular (JM-a and JM-b) and intracellular (CYT-1 and CYT-2) domains as well as 4ICD localization, and tested the relationship with clinicopathological characteristics and prognosis. The predominantly-expressed extracellular domain was JM-a, and lower CYT-2 dominance was a factor related to better relapse-free survival. CYT-2-dominance with higher nuclear 4ICD expression was a favorable prognostic marker especially in patients with the ER+ HER2- subtype treated with endocrine therapy. The absence of cytoplasmic 4ICD staining was related to better prognosis in CYT-1-dominant patients. In conclusion, analysis of splicing variants and 4ICD localization should be considered when targeting HER4 as a novel ER+/HER2- breast cancer treatment.

## INTRODUCTION

Human epidermal growth factor family receptor 4 (HER4) is a receptor tyrosine kinase and a member of the HER family, which has been reported to be associated with estrogen receptor (ER)-positive breast cancer and favorable outcome [1-3]. In contrast to the other HER family receptors, the existing evidence suggests that HER4 is characterized by anti-proliferative and pro-apoptotic activity, but relatively little is known about the activity of its functionally-distinct splicing isoforms in different clinical and biological contexts [4-6].

HER4 is composed of 3 domains, a glycosylated extracellular ligand-binding domain, a single transmembrane domain and an intracellular domain (ICD) [7], and is known to produce splicing variants. The HER4 gene undergoes alternative splicing of the extracellular domain and produces two isoforms: juxtamembrane (JM)-a, from exon 16, and JM-b from exon 15b. The two JM isoforms can be cleaved in different ways. Only the JM-a isoform has an extracellular proteolytic site [8, 9], which permits two proteolytic cleavage events by tumor necrosis factor-α converting enzyme (TACE) and γ-secretase, releasing the ectocellular domains. The resulting soluble HER4-ICD (4ICD) harbors an intrinsic nuclear localization signal and can translocate to the nucleus [10, 11]. Once in the nucleus, 4ICD is known to operate as a transcriptional cofactor, being especially potent as an ER co-activator [12, 13]. For example, estrogen promotes HER4 cleavage by enhancing TACE activity [5] and stimulates 4ICD accumulation in the nucleus [13], which promotes the expression of ER target genes such as progesterone receptor (PR) [14]. On the other hand, if cytosolic 4ICD accumulates within mitochondria, apoptosis of tumor cells is promoted through the activity of the Bcl2 homology 3 (BH3)-like proapoptotic domain of 4ICD. Thus tamoxifen is thought to impair the interaction of 4ICD with the ER, inducing 4ICD accumulation in mitochondria and leading to breast cancer cell killing [6]. However, several previous studies have shown a variety of correlations, linking cytosolic HER4 and better prognosis [15], nuclear localization of HER4 extracellular domain and worse prognosis [16], HER4 overexpression and tamoxifen resistance [17], and nuclear HER4 staining with shorter survival [18]. Consequently the prognostic value of HER4 expression in breast cancer by immunohistochemistry analysis remains controversial.

4ICD contains the intracellular cytoplasmic domain (CYT) which consists of two splicing isoforms, depending on the presence (CYT-1) or absence (CYT-2) of exon 26 which includes a consensus binding site of phosphoinositide 3-kinase (PI3-K) [8]. The two CYT isoforms have been reported to differ in ubiquitylation and kinase activity. Only CYT-1 has the PPXY motif which is necessary for ubiquitylation and thus can be degraded more easily but cannot enter the cell nucleus as easily as CYT-2 [19, 20]. In addition the existence of exon 26 of CYT-1 makes it possible for it to activate the PI3K/Akt pathway which supports not only its ability to inhibit or escape apoptosis, but also to induce chemotaxis and proliferation and reduce differentiation [21] as reported in medulloblastoma cells [22] and rat adrenal gland pheochromocytoma [8]. The CYT-1 isoform has been associated with the aggressive phenotype of medulloblastoma [22] and with poor prognosis in ovarian cancer patients [23]. Taken together, these results suggest that CYT-1 has greater oncogenic ability compared with CYT-2. Remarkably, it has recently been reported that switching from CYT-1 towards CYT-2 has an inhibitory effect on ER+ breast cancer cell growth in vitro and in vivo [24]. The clinical values of 4ICD and CYT isoforms in breast cancer remain to be elucidated. The purpose of this study was to investigate the clinical significance of HER4 splicing variants and 4ICD on prognosis, thus guiding treatment choices.

## RESULTS

### CYT-2 is dominant in the ER+ HER2- subtype, while higher cytoplasmic 4ICD staining is associated with the ER- HER2+ subtype

**Figure1:**
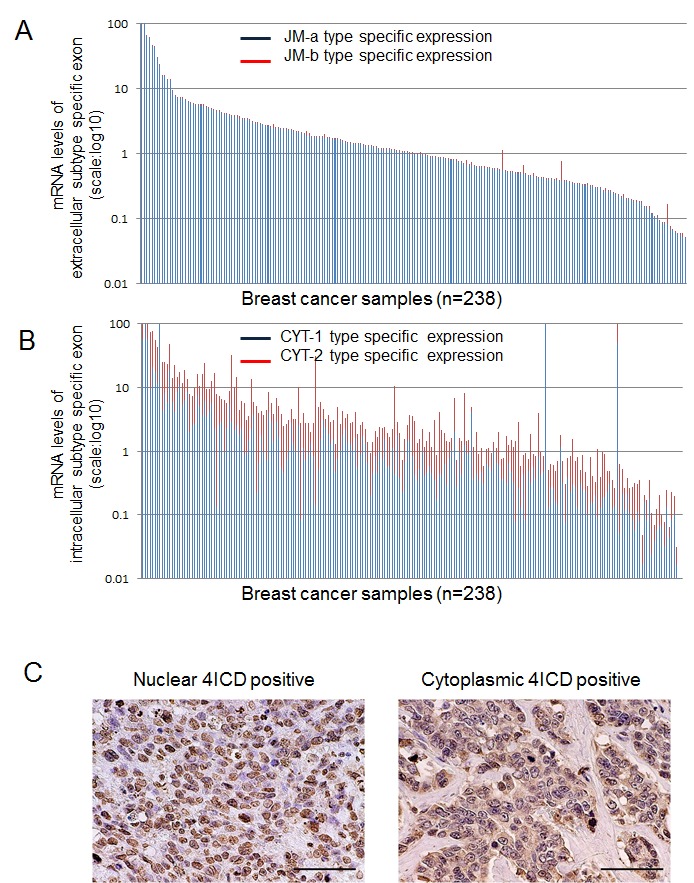
HER4 splicing isoform mRNA expression in breast cancer The samples represent 238 of breast cancer cases. Each vertical bar means one breast cancer sample. A: expression of the juxtamembrane isoforms of HER4 (JM-a: blue columns, JM-b type: red columns) B: expression of the cytoplasmic isoforms of HER4 (CYT-1: blue columns, CYT-2: red columns) C: Representative photographs of immunohistochemical staining of breast cancer sections for HER4 images using mouse monoclonal antibody HFR-1. Left: Nuclear HER4 positive staining pattern Right: Cytoplasmic HER4 positive staining pattern.

The detailed results of analysis of expression of HER4 splicing isoforms are shown Table 1, Fig 1 A, 1B and Supplementary Table 1. The median expression level was 0.95 (range: 0.002–117.94) for JM-a, 0.0043 (range: 0–4.40) for JM-b, and 1.89 × 10^4^ (range: 1–1.37×10^4^) for JM-a/JM-b. The expression level of JM-b was extremely low and close to the lower detection limit (Figure 1A), indicating that breast cancer tissues predominantly express the JM-a subtype rather than JM-b. A higher JM-a/JM-b ratio was associated with lower nuclear grade (P = 0.02), ER positivity (P = 0.005), PR positivity (P = 0.002), HER2 negativity (P = 0.001) and lower Ki67 status (P = 0.001). The JM-a/JM-b ratio was significantly higher in the ER+HER2- subtype than in the ER-HER2+ subtype (P = 0.0018; Table 1). Analysis of CYT expression revealed that median expression levels of CYT-1 were 0.55 (range: 0.001–229.79), 1.04 for CYT-2 (range: 0.002–150.45) and 0.57 for CYT-1/CYT-2 ratio (range: 0.005–597.09) (Figure 1B). Higher expressions of both CYT-1 and CYT-2 were significantly associated with lower nuclear grade, ER positivity, PR positivity and HER2 negativity (Supplementary Table 1). CYT-2 was also associated with the absence of lymph node metastasis. A lower CYT-1/CYT-2 ratio (CYT-2 dominance) was significantly associated with the absence of lymph node metastasis (P = 0.02) and ER positivity (P = 0.005). The ER+HER2- subtype had a significantly lower CYT-1/CYT-2 ratio than the ER- HER2- subtype (P = 0.038; Table 1).

**Table 1:** Association of expression ratios of HER4 spilicing isoforms with clinicopathological parameters in breast cancer

		JMa/JMb			CYT1/CYT2	
Variable	n	median (25%, 75%)	P value		median (25%, 75%)	P value
Menopause						
Pre-	67	224.34 (51.39, 496.21)	0.63		0.57 (0.33, 1.26)	0.47
Post-	171	166.54 (39.4, 461.27)			0.56 (0.34, 0.97)	
Tumor size						
≦2cm	127	200.48 (56.74, 499.71)	0.08		0.53 (0.30, 0.88)	0.09
≧2cm	111	171.56 (23.48, 422.33)			0.62 (0.38, 1.05)	
N stage						
N0	150	194.39 (44.1, 491.01)	0.32		0.51 (0.28, 0.93)	0.02*
N1-2	88	186.3 (37.18, 362.67)			0.65 (0.43, 1.09)	
Tumor grade						
Grade1	124	226.03 (56.11, 494.29)	0.002*		0.54 (0.32, 0.94)	0.09
Grade2-3	114	97 (19.11, 200.67)			0.77 (0.45, 1.09)	
ER						
Positive	194	220.13 (58.24, 494.29)	0.005*		0.53 (0.29, 0.88)	0.005*
Negative	44	54.22 (11.38, 235.33)			0.90 (0.51, 1.11)	
PR						
Positive	166	222.87 (59.05, 496.21)	0.002*		0.54 (0.32, 0.87)	0.11
Negative	72	94.38 (16.01, 254.79)			0.73 (0.39, 1.10)	
HER2						
Positive	30	56.46 (15.60, 172.58)	0.001*		0.77 (0.48, 1.10)	0.21
Negative	208	220.26 (50.44, 492.11)			0.55 (0.33, 0.96)	
Ki67						
≧15%	154	169.55 (108.91, 654.86)	0.001*		0.54 (0.34, 1.04)	0.93
<15%	80	154.13 (24.99, 354.06)			0.57 (0.34, 1)	
Subtype						
ER+ HER2-	185	220.32 (58.00, 498.67)†	0.0018†		0.53 (0.29, 0.88)‡	0.038‡
ER+ HER2+	9	177.59 (70.59, 248.23)			0.64 (0.29, 1.26)	
ER- HER2+	21	23.61 (8.47, 60.43)†			0.90 (0.51, 1.12)	
Triple negative	23	107.45 (12.36, 377.1)			0.90 (0.52, 1.12)‡	

* P value<0.05

†, ‡ Significant combination by nonparametric multiple comparison

In the immunohistochemical assessment of 4ICD, high nuclear 4ICD staining (≥ 50%) was observed in 158 patients (66%) (Fig. 1C, left), and cytoplasmic 4ICD staining (≥ 50%) was high in 146 patients (62%) (Fig. 1C, right). Both nuclear and cytoplasmic staining were observed in 84 patients (35.3%). Higher nuclear 4ICD expression was significantly associated with smaller tumor size (P = 0.02; Table 2). In contrast, higher cytoplasmic 4ICD expression was significantly associated with HER2 positivity (P = 0.0005) and PR negativity (P = 0.02; Table 2). The ER- HER2+ subtype had much higher cytoplasmic 4ICD expression than the ER+ HER2- subtype (P = 0.0094, which was almost significant under Bonferroni's correction).

**Table 2:** Association of 4ICD expression with clinicopathological parameters in breast cancer

	Nucleus				Cytoplasm		
percetage of	<50%	≧50%			<50%	≧50%	
stained cells	n (%)	n (%)	P value		n (%)	n (%)	P value
Menopause							
Pre-	30 (45%)	37 (55%)	0.38		35 (52%)	32 (48%)	0.11
Post-	67 (39%)	104 (61%)			69 (40%)	102 (60%)	
Tumor size							
≦2cm	44 (35%)	83 (65%)	0.02*		50 (39%)	77 (61%)	0.19
≧2cm	53 (48%)	58 (52%)			54 (49%)	57 (51%)	
N stage							
N0	61 (41%)	89 (59%)	0.92		66 (44%)	84 (56%)	1
N1-2	36 (41%)	52 (59%)			38 (43%)	50 (57%)	
Tumor grade							
Grade1	47 (38%)	77 (62%)	0.24		56 (45%)	68 (55%)	0.69
Grade2-3	50 (44%)	64 (56%)			48 (42%)	66 (58%)	
ER							
positive	81 (42%)	113 (58%)	0.77		90 (46%)	104 (54%)	0.09
negative	16 (36%)	28 (64%)			14 (32%)	30 (68%)	
PR							
positive	69 (41%)	97 (59%)	0.66		81 (48%)	85 (52%)	0.02*
negative	28 (39%)	44 (61%)			23 (32%)	49 (68%)	
HER2							
positive	14 (46%)	16 (54%)	0.83		6 (20%)	24 (80%)	0.0005*
negative	83 (40%)	125 (60%)			98 (47%)	110 (53%)	
Ki67							
≧15%	66 (43%)	88 (58%)	0.25		68 (44%)	86 (56%)	0.89
<15%	30 (37%)	50 (64%)			36 (46%)	44 (54%)	
CYT							
CYT-1 dominant	17 (30%)	43 (70%)	0.064		25 (42%)	35 (58%)	0.76
CYT-2 dominant	104 (58%)	74 (42%)			79 (44%)	99 (56%)	
Subtype							
ER+ HER2-	71 (38%)	114 (62%)			88 (48%)	97 (52%)†	
ER+ HER2+	4 (44%)	5 (56%)			2 (22%)	7 (78%)	
ER- HER2+	8 (38%)	13 (62%)	n.s.		4 (19%)	17 (81%)†	0.0094†
Triple negative	8 (35%)	15 (65%)			10 (43%)	13 (57%)	

* P <0.05

† Significance defined as P < 0.0083 Bonferroni's correction

### CYT-2 dominance and higher nuclear 4ICD expression are respectively better prognostic factors in ER+ HER2- patients

**Figure2:**
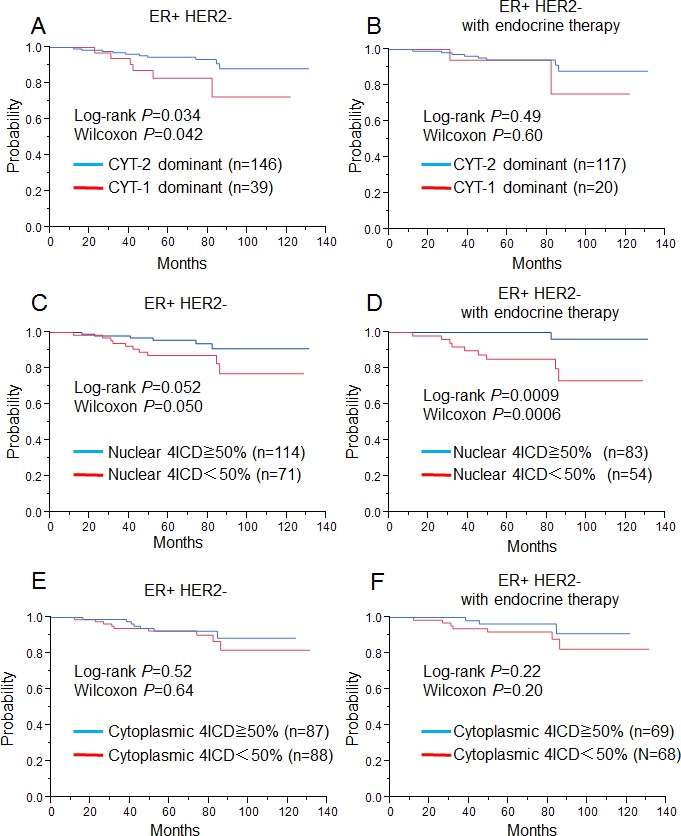
Relapse free survival for ER+ HER2- breast cancer patients (left, A, C, E: n=185) and ER+ HER2- breast cancer patients treated with only endocrine therapy (right, B, D, F: n=122), using the Kaplan-Meier methods and verified by the wilcoxon test and the log-rank test A, B: Relationship between nuclear CYT-2-dominant patients (blue line) and higher nuclear CYT-1-dominant patients (red line). C, D: between high nuclear 4ICD patients (blue line) and low nuclear 4ICD patients (red line) E, F: between high cytoplasmic 4ICD patients (blue line) and high cytoplasmic 4ICD patients (red line).

We analyzed the prognostic values of the CYT isoforms mainly because most JM isoforms were found to be of the JM-a type. We divided the expression of CYT isoforms into two groups; a CYT-1 dominant group (CYT-1/CYT-2 ≥ 1, n = 60) and a CYT-2 dominant group (CYT-1/CYT-2 < 1, n = 178). Kaplan-Meier analysis revealed that the CYT-2 dominant group was associated with better relapse-free survival (RFS) in the entire cohort (P = 0.050, by log-rank test, P = 0.056, by Wilcoxon test; Supplementary Fig. 1A). In the ER+ HER2- cohort, the CYT-2 dominant group (n = 146) was also associated with better RFS (P = 0.034: log-rank test, P = 0.042: Wilcoxon test; Fig. 2A). There was no significant difference in the cohort of ER+HER2- patients treated with endocrine therapy according to CYT dominance (Fig. 2B).

On the other hand, higher nuclear 4ICD expression (≥ 50%) was associated with better RFS in the entire cohort (P = 0.041: log-rank test, P = 0.048: Wilcoxon test; Supplementary Fig. 1B). In the ER+ HER2- cohort, higher nuclear 4ICD expression also showed a tendency of association with better RFS (P = 0.052: log-rank test, P = 0.050: Wilcoxon test; Fig. 2C). Moreover, the significance of higher nuclear 4ICD expression was enhanced in the ER+ HER2- cohort treated with endocrine therapy (P = 0.0009: log-rank test, P = 0.0006: Wilcoxon test; Fig. 2D). Cytoplasmic 4ICD expression was not associated with RFS in the entire cohort (P = 0.24: log-rank test, P = 0.13: Wilcoxon test; Supplementary Fig. 1C), either in the ER+ HER2- cohort (P = 0.52: log-rank test, P = 0.64: Wilcoxon test, Figure 2E), or in patients treated with endocrine therapy (P = 0.22: log-rank test, P = 0.20: Wilcoxon test, Fig. 2F).

### The combination of the CYT dominance and 4ICD localization could be a good prognostic marker in ER+ HER2- patients

**Figure 3:**
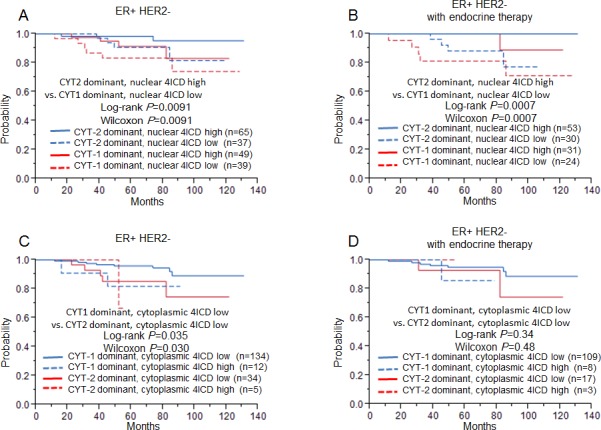
Relapse free survival for ER+ HER2- breast cancer patients (left, A, C: n=185) and ER+ HER2- breast cancer patients treated only with endocrine therapy (right, B, D: n=122), using the Kaplan-Meier methods and verified by the wilcoxon test and the log-rank test A, B: The relationship between CYT- dominance and nuclear 4ICD status. C, D: The relationship between CYT- dominance and cytoplasmic 4ICD status.

We analyzed the prognostic potential of a combination of expression of intracellular spliced variants and nuclear or cytoplasmic 4ICD (Fig. 3). Kaplan-Meier analysis revealed that the CYT-2 dominant, high nuclear 4ICD group (n = 65) showed the most favorable prognosis (5-year RFS: 98%). In contrast, the CYT1 dominant, lower nuclear 4ICD expressing patients (n = 39) exhibited the worst 5-year RFS (80%; Fig. 3A). In the ER+ HER2- cohort treated with endocrine therapy, no relapse occurred in the CYT2 dominant, higher nuclear 4ICD expressing patients (n = 53). The patients with the CYT-1 dominant, lower nuclear 4ICD phenotype (n = 24) exhibited the worst 5-year RFS (87%; Figure 3B), which showed significant survival difference (CYT2 dominant, nuclear 4ICD high vs. CYT1 dominant, nuclear 4ICD low: P = 0.0091, Fig. 3A) In the analysis for breast cancer-specific survival, higher nuclear 4ICD expression was suggested to surrogate a good prognosis (Supplementary Fig 2). Regarding CYT dominance and cytoplasmic 4ICD expression, the CYT-1-dominant group with lower cytoplasmic staining of 4ICD tended to show much better prognoses in both ER+ HER2- cohorts (Fig. 3C and D).

In the Cox's hazards model, univariate analysis revealed that the CYT-2 dominant, high nuclear 4ICD group (P = 0.019, HR: 0.23) and the CYT1-dominant, low cytoplasmic 4ICD group (P = 0.016, HR: 0.29) were both significantly associated with better prognosis in the ER+ HER2- cohort (Table 3). Multivariate analysis between these parameters and tumor size (univariate P = 0.023, HR: 3.22) and nuclear grade (univariate P = 0.046, HR 2.75) showed a significant association with better prognosis in the CYT-1 dominant and low cytoplasmic 4ICD group (P = 0.027, HR: 0.32; Table 3). In the ER+ HER2- cohort treated with endocrine therapy, higher nuclear 4ICD expression (P = 0.0007, HR: 0.071) and CYT-2 dominance (P = 0.0012, HR: 3.14×10^−10^) showed significantly better prognostic values for RFS, whereas CYT-1 dominance and low nuclear 4ICD correlated with worse prognosis (P = 0.018, HR: 4.87; Table 4). In multivariate analysis with tumor size (univariate P = 0.029, HR: 4.16), the CYT-2 dominant and high nuclear 4ICD group showed the lowest P-value of 0.0024 (HR: 3.63×10^−10^) in line with nuclear 4ICD expression (P = 0.026; HR 0.087; Table 4). The parameters associated with CYT dominance and 4ICD immunohistochemical staining had no significant association by multivariate analysis with other clinicopathological factors in the entire cohort (Supplemental Table 2).

**Table 3:** Univariate and multivariate analysis for relapse free survival in ER+ HER2- breast cancer patients (n=185)

Variable		Reference	Univariate analysis					Multivariate analysis			
			P value	HR	95% CI			P value	HR	95% CI	
					Lower	Upper				Lower	Upper
Menopause	pre vs. post	pre	0.52	0.71	0.26	2.09					
Tumor size	≦2cm vs. >2cm	≦2cm	0.023*	3.22	1.17	10.25		0.027†	3.16	1.14	10.1
Nodal status	positive vs. negative	negative	0.21	1.88	0.69	5.12					
Nuclear Grade	1 vs 2, 3	1	0.046*	2.75	1.02	8.08		0.38†	1.83	0.41	2.44
PR	positive vs. negative	negative	0.72	0.79	0.25	3.45					
Ki67	≦15% vs. >15%	≦15%	0.29	1.8	0.62	6.46					
CYT-1/CYT-2 ratio	CYT-2 dominant vs. CYT-1 dominant	CYT-2 dominant	0.056	2.85	0.97	7.71					
4ICD nuclear expression	high vs. low	low	0.056	0.38	0.13	1.03					
4ICD cytoplasmic expression	high vs. low	low	0.95	0.97	0.36	2.72					
CYT-1/CYT-2 and nuclear 4ICD	CYT-2 dominant, nuclear 4ICD high vs. others	others	0.019*	0.23	0.036	0.81		0.053‡	0.28	0.043	1.02
	CYT-2 dominant, nuclear 4ICD low vs. others	others	0.73	1.22	0.34	3.51					
	CYT-1 dominant, nuclear 4ICD high vs. others	others	0.81	1.14	0.32	3.3					
	CYT-1 dominant, nuclear 4ICD low vs. others	others	0.056	2.86	0.97	7.71					
CYT-1/CYT-2 and cytoplasmic 4ICD	CYT-1 dominant, cytoplasmic 4ICD low vs. others	others	0.016*	0.29	0.1	0.78		0.027†	0.32	0.12	0.87
	CYT-1 dominant, cytoplasmic 4ICD high vs. others	others	0.24	2.72	0.42	9.89					
	CYT-2 dominant, cytoplasmic 4ICD low vs. others	others	0.11	2.51	0.79	6.89					
	CYT-2 dominant, cytoplasmic 4ICD high vs. others	others	0.34	3.22	0.17	16.63					

* Factor showing statistical signficance in univariate analysis

† Cox proportional regression model including CYT-1 dominant, cytoplasmic HER4 negative, tumor size and Nuclear Grade

‡ Cox proportional regression model including CYT-2 dominant, nuclear HER4 positive, tumor size and Nuclear Grade

**Table 4:** Univariate and multivariate analysis for relapse free survival in ER+ HER2- breast cancer patients with endocrine therapy (n=137)

Variable		Reference	Univariate analysis					Multivariate analysis			
			P value	HR	95% CI			P value	HR	95% CI	
					Lower	Upper				Lower	Upper
Menopause	pre vs. post	pre	0.24	0.45	0.13	1.78					
Tumor size	≦2cm vs. >2cm	≦2cm	0.029*	4.16	1.15	10.35		0.12†	3.38	0.93	15.7
Nodal status	positive vs. negative	negative	0.11	2.77	0.77	10.01					
Nuclear Grade	1 vs 2, 3	1	0.15	3.68	0.55	14.99					
PR	positive vs. negative	negative	0.071	6.52x10^8^	0.83	0.83					
Ki67	≦15% vs. >15%	≦15%	0.19	2.62	0.65	17.37					
CYT-1/CYT-2 ratio	CYT-2 dominant vs. CYT-1 dominant	CYT-2 dominant	0.53	1.7	0.26	6.79					
4ICD nuclear expression	high vs. low	low	0.0007*	0.071	0.0038	0.37		0.0026‡	0.087	0.0046	0.47
4ICD cytoplasmic expression	high vs. low	low	0.21	0.44	0.094	1.58					
CYT-1/CYT-2 and nuclear 4ICD	CYT-2 dominant, nuclear 4ICD high vs. others	others	0.0012*	3.14x10^−10^	0.31	0.31		0.0024†	3.63x10^−10^	0.3	0.36
	CYT-2 dominant, nuclear 4ICD low vs. others	others	0.19	2.42	0.62	8.55					
	CYT-1 dominant, nuclear 4ICD high vs. others	others	0.38	0.43	0.024	2.32					
	CYT-1 dominant, nuclear 4ICD low vs. others	others	0.018*	4.87	1.34	17.65		0.05§	3.77	0.99	14.15
CYT-1/CYT-2 and cytoplasmic 4ICD	CYT-1 dominant, cytoplasmic 4ICD low vs. others	others	0.29	0.46	0.13	2.16					
	CYT-1 dominant, cytoplasmic 4ICD high vs. others	others	0.39	2.86	0.15	16.93					
	CYT-2 dominant, cytoplasmic 4ICD low vs. others	others	0.43	1.94	0.29	7.74					
	CYT-2 dominant, cytoplasmic 4ICD high vs. others	others	0.59	1.46x10^−8^	15.55	15.55					

* Factor showing statistical significance in univariate analysis

† Cox proportional regression model including CYT-2 dominant, nuclear HER4 high and tumor size

‡ Cox proportional regression model including HER4 nuclear expression and tumor size

§ Cox proportional regression model including CYT-1 dominant, nuclear HER4 low and tumor size

## DISCUSSION

In this study, we show that the expression ratio of two different HER4 intracellular splicing variants combined with 4ICD localization would be a potent prognostic marker in breast cancer. Patients with CYT-2 dominant tumors showed better RFS compared to those with CYT-1 dominant tumors, especially in the ER+ HER2- subtype. Nuclear 4ICD localization was also correlated with better prognosis particularly in ER+ HER2- breast cancer patients treated with endocrine therapy. The combination of these 2 characteristics, CYT-2 dominance and higher nuclear 4ICD expression, thus correlates with superior prognosis under endocrine treatment.

Junttila et al. previously analyzed expression of HER4 cleavable isoforms in 60 breast cancer specimens, and found predominance of the JM-a isoform as well as a correlation between each of higher JM-a, higher CYT-1, higher CYT-2 expression and lower histological grade, and ER/PR positivity [8, 18]. These data are absolutely consistent with our results from a larger sample size (Supplementary Table 1). In addition, Nielsen et al. reported that a lower CYT-1/CYT-2 ratio was accompanied by decreased tumor growth in ER+ breast cancer cell lines, and that a lower CYT-1/CYT-2 ratio led to the reduction of phosphorylated Akt [24]. Our prognostic analysis is in agreement with their data; a lower CYT-1/CYT-2 ratio (CYT-2 dominance) is a favorable prognostic marker in ER+ HER2- subtype cancers. We also assumed that the nuclear localization of 4ICD indicates its main site of action as the ER co-activator, and its destination in a cell is determined by the CYT isoform expressed. Naresh et al. reported that nuclear staining of 4ICD was associated with longer disease-specific survival times in tamoxifen-treated ER+ PgR+ breast cancer patients [25]. They investigated 4ICD expression in ER+ breast cancer cell lines and speculated that tamoxifen promoted mitochondrial accumulation of 4ICD and activated apoptotic proteins such as Bcl-2 homologous antagonist/killer(BAK) and Bcl-2 associated X protein(BAX) [25]. Similarly, we confirmed the enhanced prognostic significance of nuclear localization of 4ICD in the endocrine-treated ER+ HER2- subpopulation by multivariate analysis. As our endocrine-treated ER+ HER2- cohort was exposed not only to tamoxifen but also to aromatase inhibitors in over 65% of the patients, it can be assumed that the mechanism of interaction with estrogen and ER signaling considerably mediates nuclear accumulation of 4ICD. Furthermore, we reveal that the combination of CYT isoforms and localization of 4ICD can predict survival, especially for patients with CYT-2-dominant, higher nuclear 4ICD-expressing tumors in the endocrine-treated ER+ HER2- subpopulation. This conclusion is quite reasonable based on data obtained by Williams et al. and Zhu et al., showing that 4ICD is a potent ER co-activator [12, 13]. In that cohort, the opposed characteristics of CYT-1 dominance and low nuclear 4ICD expression were also elucidated as a risk factor (P = 0.050, HR: 3.77 by multivariate analysis), with significant correlations between the patients with these 2 characteristics. The expression of the ER-related genes histone deacetylases 6(HDAC6) and nuclear receptor corepressor 1(NCOR1) was up-regulated in patients with tumors showing CYT-2 dominance and higher nuclear 4ICD expression (P = 0.039 and P = 0.0097 respectively; using in-house data under submission). CYT-1 dominance and low nuclear 4ICD levels were correlated significantly with lower ER expression (P = 0.027, median, Yes vs. No: 80% vs. 90%) and higher Ki67 labeling index (P = 0.041, median, Yes vs. No: 0.22 vs.0.16; Supplementary Fig. 3), as well as up-regulation of MYC gene expression (data not shown); all of which are endocrine-resistant features. Taken together, these data lead to the hypothesis that a tumor with nuclear translocation of CYT-2-dominant 4ICD may reinforce ER-dependent growth in cooperation with ER transcriptional activity, which implies that these characteristics would be a useful index when choosing endocrine mono-therapy for ER+ HER2- patients.

On the other hand, a higher CYT-1/CYT-2 ratio (CYT-1 dominance) correlated with the most aggressive breast cancer subtypes such as Triple Negative (TN), and CYT-1 dominant, with patients having a worse prognosis even in the ER+ HER2- population. One report in ovarian cancer showed that a higher CYT-1/CYT-2 ratio is associated with older age, higher tumor grade, larger residual tumor size, abnormal p53 value and higher Ki67 index [23]. These may be partially supported by the evidence that CYT-1 includes a PI3K binding site and can activate the PI3K/Akt pathway leading to tumor progression [21]. However, there have been some contradictory reports regarding CYT functions in mammary epithelial cells. Overexpression of CYT-1 is thought to promote epithelial differentiation but decrease cell growth, but overexpression of CYT-2 increases proliferation and promotes hyperplasia [26]. This CYT-1-specific suppressive characteristic requires an intact PPXY sequence which is responsible for its ubiquitylation. Although the cleaved intracellular domain CYT-2 was reported to translocate into the nucleus more readily than CYT-1 [18, 20], our results show that CYT-1-dominant tumors express higher nuclear staining of 4ICD compared to cytoplasmic staining (P = 0.064; Table 2). Moreover, the population of ER-negative patients with CYT-1 dominance and high nuclear 4ICD was significantly greater compared with the ER-positive population (ER positive vs. ER negative: 27% vs. 54%; P = 0.0007, data not shown). We speculate that loss of the PPXY sequence leading to accumulation of abnormal CYT-1 in the nucleus might contribute to the dominant-negative effect and result in the aggressive cancer phenotype. In the TN and HER2+ subtypes, Machleidt et al. recently analyzed the CYT-1/CYT-2 ratio and reported that there was no prognostic stratification of CYT isoforms for these subtypes [27]. Although our population is smaller than their cohort, CYT-1 dominant patients tended to show worse prognosis in the HER2+ subtype cohort (P = 0.15 by log-rank test; Supplementary Fig.4A). It is worthy of note, that cytoplasmic 4ICD was highly expressed in the ER- HER2+ subtype (Table 2) and had some association with worse prognosis in the HER2+ subpopulation (P = 0.058; Supplementary Fig. 4C). A reasonable explanation is that a signal transduction system such as the PI3K-Akt pathway, typically present in HER2+ tumors, could be affected by the CYT-1 isoform of 4ICD if it is located in the cytoplasm. As evidence of this, patients with CYT-1 dominance but low cytoplasmic 4ICD showed significantly better prognosis in the ER+HER2- cohort (P = 0.027 by multivariate model; Table 3). This significance disappeared in the clinician's selected endocrine-treated cohort which was inferred to have less signal transduction activity other than ER-signaling (P = 0.29 by univariate model; Table 4). All these data suggest that HER4 could be a potent therapeutic target; a HER4 JM-a type-specific anti-HER4 monoclonal antibody 1479 is available which has been reported to block HER4 cleavage in breast cancer cells and to suppress breast cancer cell growth both in vivo and in vitro [5, 28]. In addition, the splice-switching oligonucleotide technique, which converts CYT-1 to CYT-2, has been reported to inhibit MCF-7 cell growth [24]. Of course that our analysis has some limitations; the evaluation of the presence of activating ligands or dimerization partners which could affect HER4 function would be required.

According to our results, patients other than those with CYT-2 dominant and higher nuclear 4ICD expression may receive some benefit from use of these therapeutic strategies, which would be more beneficial if used together with endocrine therapy for ER+ HER2- breast cancer. Future studies will hopefully elucidate suitable approaches to the targeting of HER4 in clinical breast cancer therapy.

## PATIENTS AND METHODS

### Patients and breast tissues

Breast tumor specimens from 238 patients with invasive breast cancer, who were treated at Kumamoto University Hospital between 2001 and 2009, were included in this study. The patients were from a consecutive series, and excluded those with other malignancies or bilateral breast cancer. Samples were snap-frozen in liquid nitrogen at the time of pre-therapeutic biopsy or surgical treatment. Samples were stored at −80°C until simultaneous total RNA and genomic DNA extraction. Informed consents were obtained from all patients. The ethics committee of Kumamoto University Graduate School of Medical Sciences approved the study protocol. The median age of the patients was 58 years old (range, 30–93). Adjuvant and neoadjuvant treatment were administered in accordance with the recommendations of the St. Gallen international expert consensus on the primary therapy of early breast cancer. Of the 238 patients, 145 were treated only with endocrine therapy; 13 received tamoxifen, 24 patients were treated with tamoxifen + LHRH analogue, 9 patients were given toremifen, 79 received anastrozole, 5 received letrozole, and 15 were treated with exemestane. Patients were followed postoperatively every three months. The median follow-up period was 65 months (range, 3–133).

### Real-time quantitative reverse transcription polymerase chain reaction analysis

Total RNA was isolated from tissue specimens using the Allprep DNA/RNA Mini Kit (Qiagen, Valencia, CA, U.S.A.). Total RNA (0.5 μg) was reverse transcribed to cDNA using the PrimeScript RT reagent Kit (Takara Bio Inc., Otsu, Japan), according to the manufacturer's protocol.

Real-time quantitative reverse transcription polymerase chain reaction (RT-qPCR) was used to assess HER4 mRNA expression. Real-time RT-qPCR was carried out in a solution containing 10.0 μL of 2× TaqMan® Fast Universal PCR Master Mix (4367846, Applied Biosystems), 1.0 μL of TaqMan® Gene Expression Assay (HER4:Hs00171783_m1, β-Actin: Hs01060665_g1, PUM1:Hs00982775_m1, TAF10:Hs00359540_g1, Applied Biosystems), 7.5 μL of nuclease-free water and 1.5 μL of cDNA sample (10 ng/μL) in a final volume of 20 μL.

HER4 is alternatively spliced and variants in the extracellular juxtamembrane domain or intracellular domain are produced [8]. To investigate these spliced variants, probes for JM-a or JM-b were designed to hybridize to each isoform-specific exon (exon 16 or exon 15 respectively), and probes for CYT-1 or CYT-2 were also designed to hybridize to the isoform-specific regions (exon 26 or the junction between exons 25 and 27 respectively), according to a previous report [29]. The primers were synthesized based on exon sequences flanking the site recognized. The primer/probe sets were as follows;

HER4 JM-a/JM-b 5'-primer: 5'-CCACCCATGCCATCCAAA-3',

HER4 JM-a/JM-b 3'primer: 5'-CCAATTACTCCAGCTGCAATCA-3',

HER4 JM-a probe: 5'-ATGGACGGGCAATTCCACTTTACCA-3',

HER4 JM-b probe: 5'-CTCAAGTATTGAAGACTGCATCGGCCTGAT-3',

HER4 CYT-1/CYT2 5'-primer: 5'- CAACATCCCACCTCCCATCTATAC-3',

HER4 CYT-1/CYT2 3'-primer: 5'-ACACTCCTTGTTCAGCAGCAAA-3',

HER4 CYT-1 probe: 5'-TGAAATTGGACACAGCCCTCCTCCTG-3',

HER4 CYT-2 probe: 5'-AATTGACTCGAATAGGAACCAGTT TGTATACCGAGAT-3'.

The efficacy, sensitivity and specificity of these primer/probe sets in distinguishing the isoforms was previously confirmed [29]. cDNA at the same concentration showed similar Ct values between JM-a and JM-b, or between CYT-1 and CYT-2 using our chosen primer/probe sets [29].

Thermal cycling was performed in an ABI PRISM 7500. Negative controls were included in each run. Relative mRNA levels were determined from the threshold cycle for amplification using the Δ ΔCt method. Determination of Ct values was performed in duplicate and normalized to the Ct values of simultaneous duplicate measurements of the expression of 3 housekeeping genes –β-Actin, PUM1, TAF10 –from the same samples.

### Immunohistochemistry and image analysis

Immunohistochemical staining was performed using the avidin-biotin complex method. Histological sections (4 μm) were deparaffinized and then rehydrated. The sections were incubated for 10 min in methanol containing 0.3% hydrogen peroxide to block endogenous peroxidase, then microwaved for antigen retrieval. After nonspecific staining had been blocked using a blocking agent, sections were incubated with the primary antibody at 4 μg/mL diluted in Histofine Simple Stain MAX-PO^®^ (Nichirei, Tokyo, Japan) at room temperature for 60 min. HER4 was detected using the secondary mouse monoclonal antibody HFR-1 (Neomarker, Fremont, CA), which recognizes the intracellular domain of HER4. Detection was completed by incubation with a 3, 3' diaminobenzidine (DAB) solution. Finally the sections were counterstained with hematoxylin, dehydrated and mounted. 4ICD staining was categorized by the number of stained cells as a percentage of all cancer cells.

Mouse monoclonal antibodies were used for detection of ER (1D5, 1:50; Dako Japan), PR (PgR636, 1:800; Dako Japan), Human epidermal growth factor receptor 2(HER2) (1:200; Dako Japan, Tokyo, Japan) and Ki67 (MIB-1, 1:50; Dako Japan). ER and PR status were considered positive when there was ≥1% of nuclear staining. Membranous immunostaining for HER2 was evaluated and scored on a scale of 0 to 3+. Tumors with scores of 3+ (defined as uniform intense membrane staining of >30% of tumor cells) or with a ≥2.2-fold increase in HER2 gene amplification as determined by fluorescence in situ hybridization were considered to be positive for HER2 overexpression. Ki67 was scored for the percentage of nuclear staining cells out of all cancer cells in the invasive front of the tumor (Ki67 labeling index).

### Statistical analysis

The associations of HER4 immunohistochemical staining patterns with clinicopathological features were analyzed using the chi-square test, and Bonferroni's correction was applied for multiple comparisons with subtypes for which P < 0.0083. The significance of differences in expression levels of HER4 spliced variants was tested by the Wilcoxon test. The Steel-Dwass test was used to analyze the difference between serial parameters by RT-qPCR and tumor subtypes. Relapse free survival curves were generated using the Kaplan-Meier method and verified by the Wilcoxon test and log-rank test. Cox's proportional hazards model was used for the univariate and multivariate analysis of prognostic values. Statistical significance was defined as P < 0.05 except for data under Bonferroni's correction. JMP software version 10.0.1 for Windows (SAS institute Japan, Tokyo, Japan) was used for statistical analysis.

## SUPPLEMENTARY FIGURES AND TABLES





## References

[1] Fujiwara S, Ibusuki M, Yamamoto S, Yamamoto Y, Iwase H (2012). Association of ErbB1-4 expression in invasive breast cancer with clinicopathological characteristics and prognosis. Breast Cancer.

[2] Sundvall M, Iljin K, Kilpinen S, Sara H, Kallioniemi OP, Elenius K (2008). Role of ErbB4 in breast cancer. J Mammary Gland Biol Neoplasia.

[3] Pawlowski V, Revillion F, Hebbar M, Hornez L, Peyrat JP (2000). Prognostic value of the type I growth factor receptors in a large series of human primary breast cancers quantified with a real-time reverse transcription-polymerase chain reaction assay. Clin Cancer Res.

[4] Ghayad SE, Vendrell JA, Ben Larbi S, Dumontet C, Bieche I, Cohen PA (2010). Endocrine resistance associated with activated ErbB system in breast cancer cells is reversed by inhibiting MAPK or PI3K/Akt signaling pathways. Int J Cancer.

[5] Hollmen M, Liu P, Kurppa K, Wildiers H, Reinvall I, Vandorpe T, Smeets A, Deraedt K, Vahlberg T, Joensuu H, Leahy DJ, Schoffski P, Elenius K (2012). Proteolytic processing of ErbB4 in breast cancer. PLoS One.

[6] Naresh A, Long W, Vidal GA, Wimley WC, Marrero L, Sartor CI, Tovey S, Cooke TG, Bartlett JM, Jones FE (2006). The ERBB4/HER4 intracellular domain 4ICD is a BH3-only protein promoting apoptosis of breast cancer cells. Cancer Res.

[7] Vidal GA, Naresh A, Marrero L, Jones FE (2005). Presenilin-dependent gamma-secretase processing regulates multiple ERBB4/HER4 activities. J Biol Chem.

[8] Veikkolainen V, Vaparanta K, Halkilahti K, Iljin K, Sundvall M, Elenius K (2011). Function of ERBB4 is determined by alternative splicing. Cell Cycle.

[9] Maatta JA, Olli K, Henttinen T, Tuittila MT, Elenius K, Salmivirta M (2009). Removal of cell surface heparan sulfate increases TACE activity and cleavage of ErbB4 receptor. BMC Cell Biol.

[10] Muraoka-Cook RS, Feng SM, Strunk KE, Earp HS (2008). ErbB4/HER4: role in mammary gland development, differentiation and growth inhibition. J Mammary Gland Biol Neoplasia.

[11] Ni CY, Murphy MP, Golde TE, Carpenter G (2001). gamma -Secretase cleavage and nuclear localization of ErbB-4 receptor tyrosine kinase. Science.

[12] Williams CC, Allison JG, Vidal GA, Burow ME, Beckman BS, Marrero L, Jones FE (2004). The ERBB4/HER4 receptor tyrosine kinase regulates gene expression by functioning as a STAT5A nuclear chaperone. J Cell Biol.

[13] Zhu Y, Sullivan LL, Nair SS, Williams CC, Pandey AK, Marrero L, Vadlamudi RK, Jones FE (2006). Coregulation of estrogen receptor by ERBB4/HER4 establishes a growth-promoting autocrine signal in breast tumor cells. Cancer Res.

[14] Rokicki J, Das PM, Giltnane JM, Wansbury O, Rimm DL, Howard BA, Jones FE (2010). The ERalpha coactivator, HER4/4ICD, regulates progesterone receptor expression in normal and malignant breast epithelium. Mol Cancer.

[15] Thor AD, Edgerton SM, Jones FE (2009). Subcellular localization of the HER4 intracellular domain, 4ICD, identifies distinct prognostic outcomes for breast cancer patients. Am J Pathol.

[16] Tovey SM, Dunne B, Witton CJ, Cooke TG, Bartlett JM (2006). HER4 in breast cancer: comparison of antibodies against intra- and extra-cellular domains of HER4. Breast Cancer Res.

[17] Guler G, Iliopoulos D, Guler N, Himmetoglu C, Hayran M, Huebner K (2007). Wwox and Ap2gamma expression levels predict tamoxifen response. Clin Cancer Res.

[18] Junttila TT, Sundvall M, Lundin M, Lundin J, Tanner M, Harkonen P, Joensuu H, Isola J, Elenius K (2005). Cleavable ErbB4 isoform in estrogen receptor-regulated growth of breast cancer cells. Cancer Res.

[19] Sundvall M, Korhonen A, Paatero I, Gaudio E, Melino G, Croce CM, Aqeilan RI, Elenius K (2008). Isoform-specific monoubiquitination, endocytosis, and degradation of alternatively spliced ErbB4 isoforms. Proc Natl Acad Sci U S A.

[20] Sundvall M, Peri L, Maatta JA, Tvorogov D, Paatero I, Savisalo M, Silvennoinen O, Yarden Y, Elenius K (2007). Differential nuclear localization and kinase activity of alternative ErbB4 intracellular domains. Oncogene.

[21] Kainulainen V, Sundvall M, Maatta JA, Santiestevan E, Klagsbrun M, Elenius K (2000). A natural ErbB4 isoform that does not activate phosphoinositide 3-kinase mediates proliferation but not survival or chemotaxis. J Biol Chem.

[22] Ferretti E, Di Marcotullio L, Gessi M, Mattei T, Greco A, Po A, De Smaele E, Giangaspero F, Riccardi R, Di Rocco C, Pazzaglia S, Maroder M, Alimandi M, Screpanti I, Gulino A (2006). Alternative splicing of the ErbB-4 cytoplasmic domain and its regulation by hedgehog signaling identify distinct medulloblastoma subsets. Oncogene.

[23] Paatero I, Lassus H, Junttila TT, Kaskinen M, Butzow R, Elenius K (2013). CYT-1 isoform of ErbB4 is an independent prognostic factor in serous ovarian cancer and selectively promotes ovarian cancer cell growth in vitro. Gynecol Oncol.

[24] Nielsen TO, Sorensen S, Dagnaes-Hansen F, Kjems J, Sorensen BS (2013). Directing HER4 mRNA expression towards the CYT2 isoform by antisense oligonucleotide decreases growth of breast cancer cells in vitro and in vivo. Br J Cancer.

[25] Naresh A, Thor AD, Edgerton SM, Torkko KC, Kumar R, Jones FE (2008). The HER4/4ICD estrogen receptor coactivator and BH3-only protein is an effector of tamoxifen-induced apoptosis. Cancer Res.

[26] Muraoka-Cook RS, Sandahl MA, Strunk KE, Miraglia LC, Husted C, Hunter DM, Elenius K, Chodosh LA, Earp HS (2009). ErbB4 splice variants Cyt1 and Cyt2 differ by 16 amino acids and exert opposing effects on the mammary epithelium in vivo. Mol Cell Biol.

[27] Machleidt A, Buchholz S, Diermeier-Daucher S, Zeman F, Ortmann O, Brockhoff G (2013). The prognostic value of Her4 receptor isoform expression in triple-negative and Her2 positive breast cancer patients. BMC Cancer.

[28] Hollmen M, Maatta JA, Bald L, Sliwkowski MX, Elenius K (2009). Suppression of breast cancer cell growth by a monoclonal antibody targeting cleavable ErbB4 isoforms. Oncogene.

[29] Junttila TT, Laato M, Vahlberg T, Soderstrom KO, Visakorpi T, Isola J, Elenius K (2003). Identification of patients with transitional cell carcinoma of the bladder overexpressing ErbB2, ErbB3, or specific ErbB4 isoforms: real-time reverse transcription-PCR analysis in estimation of ErbB receptor status from cancer patients. Clin Cancer Res.

