# Supplementary material for: The localization of HER4 intracellular domain and expression of its alternately-spliced isoforms have prognostic significance in ER+ HER2- breast cancer
Source: Oncotarget. 2014 May 28;5(11):3919–30. doi: 10.18632/oncotarget.2002 (PMC4116531; doi:10.18632/oncotarget.2002)
Supplement: Supplementary file 1 [file oncotarget-05-3919-s001.pdf]

The localization of HER4 intracellular domain and expression of its alternately-spliced isoforms have prognostic significance in ER+ HER2- breast cancer - Fujiwara et al

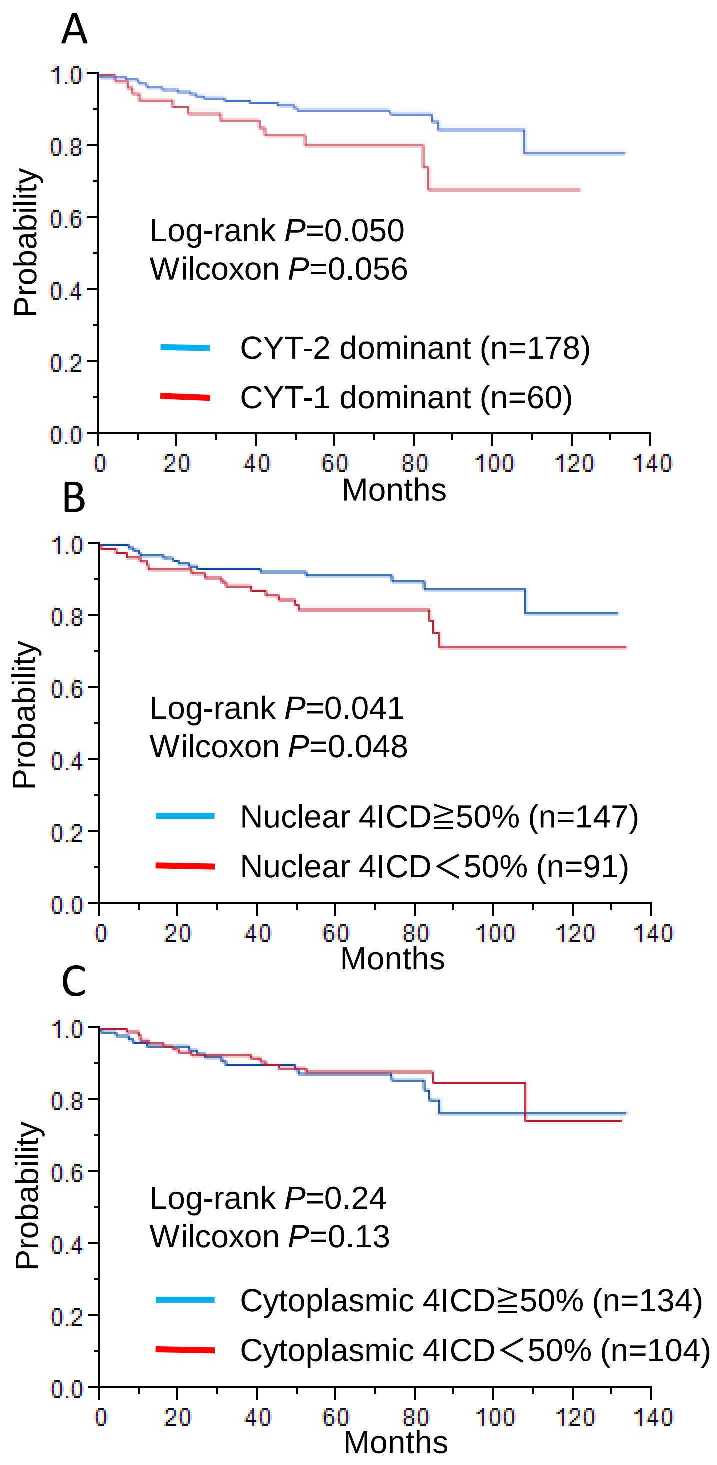

Supplementary figure 1. Relapse-free survival according to dominance of CYT isoform (A), Nuclear 4ICD expression (B) and cytoplasmic 4ICD expression (C) for the entire cohort (n = 238).

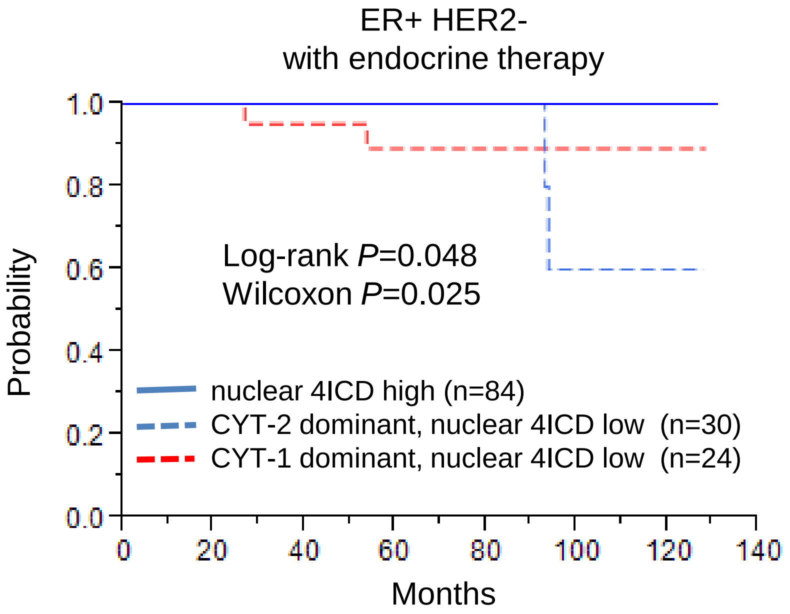

Supplementary figure 2. Breast cancer-specific survival according to the combination of dominance of CYT isoform and Nuclear 4ICD expression for endocrine-treated ER+HER2- breast cancer patients (n = 84).

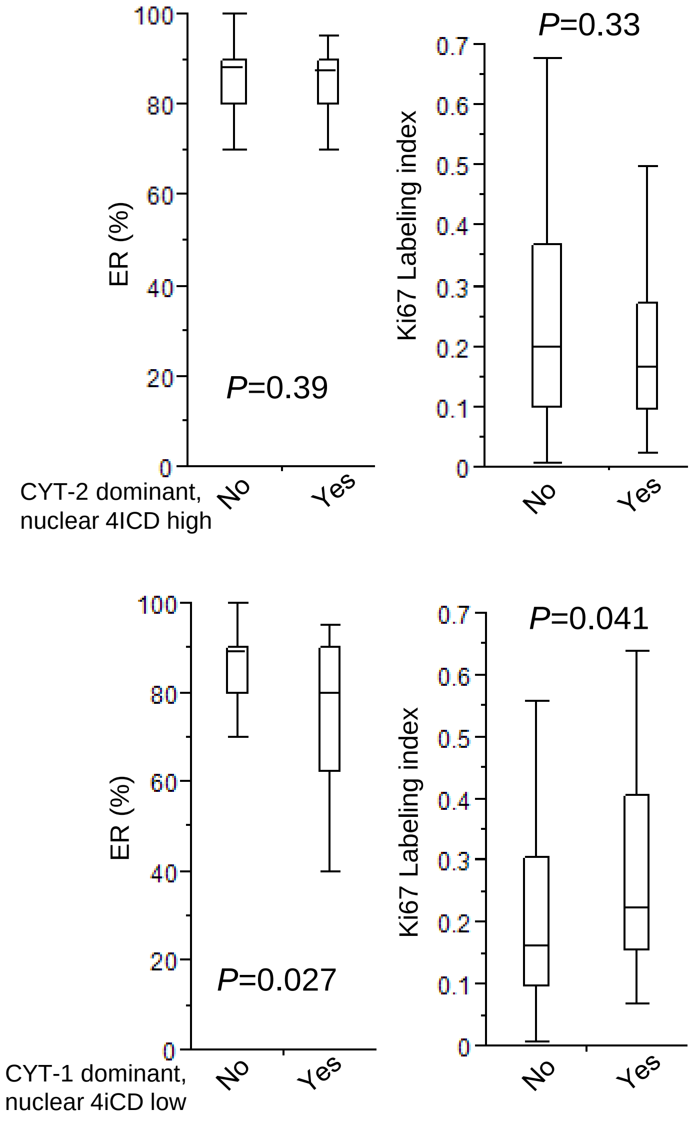

Supplementary figure 3. Correlation between CYT dominance / 4ICD localization status, ER expression, and Ki67 in ER+ HER2- breast cancer patients treated with endocrine therapy (n = 145). CYT-2 dominant, high nuclear 4ICD-expressed patients showed no correlation (upper); otherwise CYT-1 dominant, low nuclear 4ICD-expressed patients showed lower ER positivity and higher Ki67 labeling index (lower).

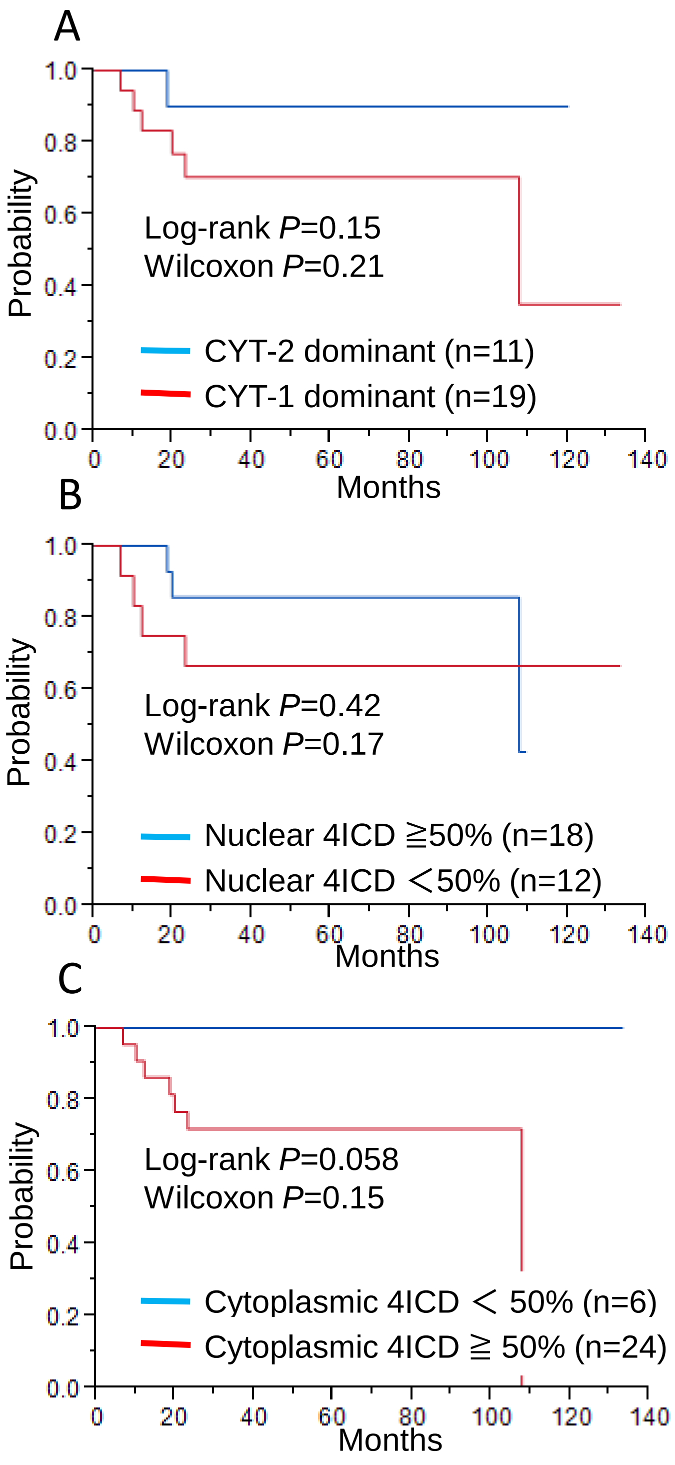

Supplementary figure 4. Relapse-free survival according to dominance of CYT isoform (A), Nuclear 4ICD expression (B) and cytoplasmic 4ICD expression (C) for HER2+ breast cancer patients (n = 30).
